# Ticks and Tick-Borne Pathogens Encountered by Dogs and Cats: A North European Perspective

**DOI:** 10.1155/tbed/5574554

**Published:** 2025-06-04

**Authors:** Jani J. Sormunen, Eero J. Vesterinen, Tero Klemola

**Affiliations:** Department of Biology, University of Turku, Turku, Finland

**Keywords:** *Borrelia*, cats, dogs, pets, TBEV, ticks, tick-borne pathogens

## Abstract

Climate change is increasing the worldwide burden of tick-borne diseases (TBDs). Dramatic increases in human cases of borreliosis have been reported during the past few decades, including from Finland, located in North Europe. As human exposure to ticks carrying pathogens is increasing, so likely is exposure of dogs and cats. However, feline or canine TBD cases are not notifiable. Likewise, no combined databases of cases exist in Finland, hindering assessment of related trends. Here, we utilize crowdsourced tick samples to reveal how commonly and to which species of TBPs dogs and cats are exposed locally. *Borrelia* spp., *Rickettsia* spp., *Anaplasma phagocytophilum*, *Neoehrlichia mikurensis*, *Babesia* spp., *Francisella tularensis*, *Bartonella* spp., and tick-borne encephalitis virus (TBEV) were screened using qPCR from a total of 3697 *Ixodes ricinus* and 2355 *Ixodes persulcatus* removed from dogs and cats. Furthermore, the spatial occurrence of the screened pathogens was mapped on the national level. An overwhelming majority (99%) of ticks removed from dogs and cats were adults. Prevalence rates in adult ticks were 26.2% for *Borrelia*, 9.3% for *Rickettsia*, 1.1% for *A. phagocytophilum*, 1.1% for TBEV, 0.6% for *N. mikurensis*, and 0.4% for *Babesia*. *Bartonella* and *F. tularensis* were not detected. All detected pathogens were observed from ticks removed from both host species and both tick species. *Borrelia* and *Rickettsia* were detected from every Finnish administrative region, whereas the occurrence of other pathogens was sporadic. This study shows that dogs and cats in Finland are frequently exposed to ticks and TBPs, highlighting that methods for protecting the animals from ticks should be further promoted. The ticks removed from dogs and cats were almost exclusively adult ticks, despite juvenile life stages being more numerous in nature. This raises questions about the numbers of juvenile ticks successfully feeding on dogs and cats and how dogs and cats are thus potentially contributing to tick population upkeep.

## 1. Introduction

Ticks and tick-borne diseases (TBDs) form a substantial threat to human and pet welfare across the world. During recent years, some hundreds of thousands of human TBD cases have been reported annually in Europe [1]. Particularly the disease burden of Lyme borreliosis has increased dramatically [2]. Following this trans-European trend, the numbers of both Lyme borreliosis and tick-borne encephalitis (TBE) cases have been increasing also in Finland [3], with new peak numbers of human cases reported for both in 2023. As human exposure to ticks carrying tick-borne pathogens (TBPs) appears to be increasing, so likely is pet exposure.

Two tick species commonly bite humans and pets in Finland, the sheep tick (*Ixodes ricinus*) and the taiga tick (*Ixodes persulcatus*) [4]. Previous studies conducted in Finland have shown that both dogs and cats are common hosts for ticks, with over 80% of roughly 20,000 ticks received via a nationwide citizen science campaign in 2015 being collected from dogs and cats [4]. Likewise, 59.5% out of ~78,000 tick observations made to an online tick surveillance website in 2021 (Punkkilive; www.punkkilive.fi/en) were reported from dogs or cats [5]. Finally, a recent study conducted at veterinary clinics in Finland revealed TBPs in 17% of ticks collected from dogs and cats [6]. Unfortunately, unlike for human cases, feline or canine TBD cases are not notifiable and no comprehensive and/or open databases for these cases exist in Finland. As such, data on canine and feline cases are not readily available, complicating the assessments of related trends.

Members of several groups of TBPs capable of infecting dogs and cats have been reported from ticks in Finland. The most commonly detected pathogens are bacteria of the *Borrelia burgdorferi* sensu lato (henceforth BBSL) group, causing Lyme borreliosis in humans [7–9]. Approximately one fifth of questing *I. ricinus* and *I. persulcatus* nymphs and one third of questing adults carry BBSL locally [7–12]. Overall, clinical manifestations are more commonly reported from dogs than cats [13, 14]. However, while relatively high seroprevalence has been reported for particularly dogs [15–17], seropositive animals commonly appear asymptomatic. It is currently unknown if symptomatic disease cases are linked to specific species/strains of BBSL. In any case, pets are likely encountering more ticks carrying BBSL due to rising tick numbers [8]. As such, more symptomatic cases may be expected as exposure rates increase.

Bacteria of the genus *Rickettsia* are typically the second most common pathogens detected in ticks in Finland. They appear to be more common in urban than peri-urban or rural environments locally, with approximately one in 10 nymphs carrying the pathogens in urban green spaces [7, 8, 10, 11, 18, 19]. Feline disease cases caused by tick-borne species *R. conorii* and *R. massiliae* have been reported from Europe [20, 21], as well as canine cases caused by *R. ricketsii* and *R. conorii* [21, 22]. As far as we are aware, no human, feline, or canine cases of tick-borne rickettsiosis have ever been reported from Finland.

*Anaplasma phagocytophilum* and *Neoehrlichia mikurensis* are pathogenic bacteria from the family Anaplasmataceae that have also consistently been detected in questing *I. ricinus* and *I. persulcatus* in Finland, although at markedly lower prevalence than BBSL or *Rickettsia* [7, 9, 10, 23]. Cases of canine and feline anaplasmosis are caused by *A. phagocytophilum* and *A. platys* in Europe [24], the latter of which has not been reported from ticks in Finland. As with borreliosis, feline cases appear to be less common than canine, although both can be considered rare [24, 25]. *N. mikurensis* is a novel, zoonotic TBP that has emerged in Europe and Asia in recent decades [26]. For humans, disease cases have most commonly been reported in immunocompromised patients, but some also from previously healthy people [26, 27]. No feline cases have been reported, whereas cases of canine neoehrlichiosis have been reported from Germany and Switzerland [28, 29]. While disease cases currently appear rare, it is possible that the pathogen has only recently emerged in tick populations in Finland and elsewhere [10]. As such, it may become more common in the future, increasing human, feline, and canine exposure to infected ticks and, consequently, disease cases.

*Babesia* spp. are protozoan parasites primarily transmitted by ticks, with worldwide economic, veterinary, and medical impact [30]. Over 20 species of *Babesia* pathogenic to domestic animals have been identified, with at least seven species linked to canine and four species to feline babesiosis [31, 32]. Species causing feline babesiosis are not found in Europe, and feline cases therein are correspondingly extremely rare and imported [31]. For species associated with dogs, *Ba. vogeli*, *Ba. gibsoni* and *Ba. canis* are found in Europe, with most disease cases caused by *Ba. canis* [32]. *Babesia canis* is mainly vectored by *Dermacentor reticulatus* ticks, which have not yet been detected from Finland. Correspondingly, while cases of canine babesiosis have been reported from Finland, there is no conclusive evidence that any have been acquired from within the country [33, 34]. However, northward expansion of *D. reticulatus* has been observed in recent years, which may also provide novel opportunities for introduction via, for example, migrating birds [35, 36].

The TBE virus (TBEV) has become more common in Finland during the past few decades. This has been seen in both the increasing numbers and geographical distribution of TBE cases [37], as well as novel virus foci detected based on screening of ticks and reservoir hosts [10, 38]. Consequently, not only humans but also dogs and cats are likely exposed to the virus more commonly. However, cats appear not to suffer clinical manifestations from TBEV infections. While clinical manifestations appear quite rare for dogs as well (relative to the high exposure dogs have) [39], severe cases and mortality have also been reported [40]. Due to their tendency to roam around in vegetation and collect ticks effectively, the use of dogs as sentinels for TBEV has been considered [41, 42]. However, due to the presence of also severe cases of TBE and the possibility of using other species as sentinels [43], protecting dogs from ticks should take priority.

While data exist on the prevalence rates of several TBPs in questing ticks in Finland [7, 8, 11, 12, 18, 44–53], there is only limited information regarding the pathogens encountered by dogs and cats or the rates of these encounters [6]. In an effort to gain insight into tick and TBP exposure of dogs and cats in Finland, we utilized samples from a citizen science campaign organized by the University of Turku tick project (www.puutiaiset.fi) in 2015 [4] to analyze the diversity and prevalence of TBPs found from *I. ricinus* and *I. persulcatus* ticks removed from dogs and cats. Likewise, we looked at differences in the spatial occurrence of these pathogens at the administrative region and municipality levels.

## 2. Materials and Methods

### 2.1. Tick Data

Tick samples from a citizen science campaign organized by the University of Turku tick project in 2015 were utilized in this study [4, 9]. In the campaign, researchers asked the public to send ticks to the University of Turku, where they were morphologically identified to species level and life stage [4]. In total, 14,889 ticks removed from cats and dogs that could be identified to the species level were received via the letters.

### 2.2. Laboratory Analyses

**Figure 1:**
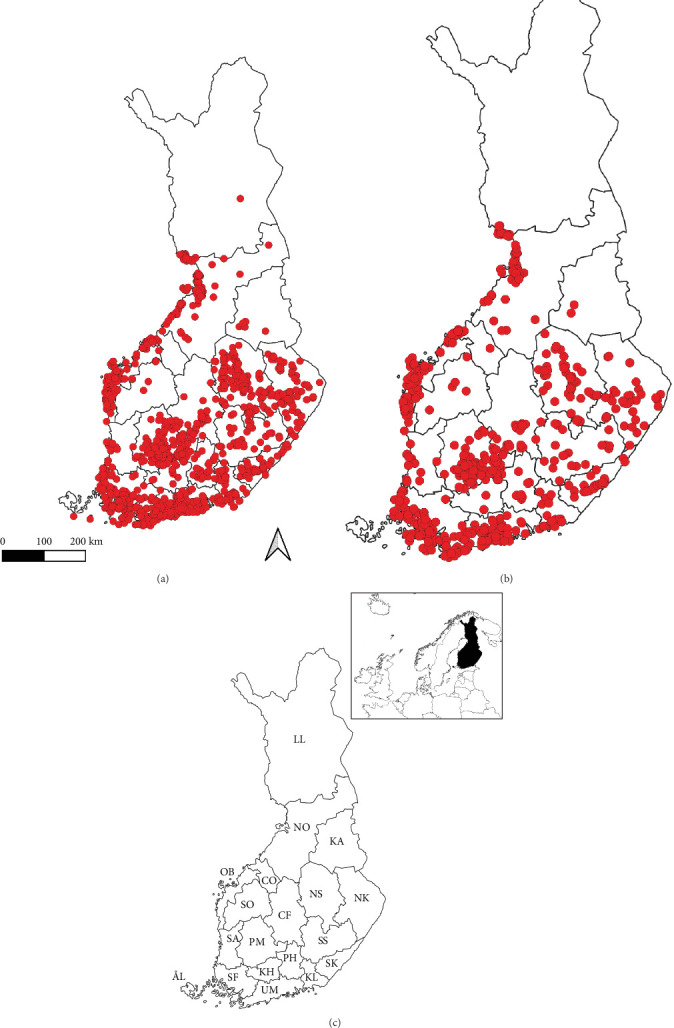
Geographical locations of analyzed tick samples removed from dogs (A) and cats (B). Finnish administrative regions are presented in panel (C): ÅL, Åland; CF, Central Finland; CO, Central Ostrobothnia; KA, Kainuu; KH, Kanta-Häme; KL, Kymenlaakso; LL, Lapland; NK, North Karelia; NO, North Ostrobothnia; NS, North Savo; OB, Ostrobothnia; PH, Päijät-Häme; PM, Pirkanmaa; SA, Satakunta; SF, Southwest Finland; SK, South Karelia; SO, South Ostrobothnia; SS, South Savo; UM, Uusimaa.

Out of the 14,889 ticks from dogs and cats, 6081 were screened for the presence of *Borrelia* spp., *A. phagocytophilum*, *Rickettsia* spp., *Babesia* spp., *N. mikurensis*, *F. tularensis*, *Bartonella* spp., and TBEV (Figure 1). Only a subset of the citizen science samples could be analyzed due to financial restraints. Since the majority of the citizen science samples were *I. ricinus* from southern Finland and collected from dogs [4], we did not perform random sampling. Instead, we aimed to collect a representative set of study samples to enable robust statistical testing. Thus, samples were selected to roughly equally represent both tick species (*I. ricinus* and *I. persulcatus*), hosts of interest (dogs and cats), and the major collection areas of the whole collection. Out of the 6081 samples, 1873 were gathered from samples analyzed for previous studies [4, 9]. For the remaining 4208 samples, total DNA and RNA were extracted from ticks using NucleoSpin 96 RNA kits and RNA/DNA buffer sets (Macherey-Nagel, Germany), following the kit protocols (NucleoSpin 96 RNA Core Kit: Rev. 05/April 2014 and RNA/DNA buffer set: Rev. 09/April 2017). DNA extracts were stored at −20°C and RNA samples at −80°C to await further analyses.

The listed pathogens were screened from DNA or RNA (TBEV) extracts using quantitative real-time PCR (qPCR; reverse transcription qPCR for TBEV). Multiplexed assays were used for screening of *A. phagocytophilum*, *Babesia* spp., and *N. mikurensis* (multiplex 1), and *Rickettsia* spp., *Bartonella* spp., and *F. tularensis* (multiplex 2) to save time and reagents [12]. The primers “Bb23S” [54], originally designed for screening BBSL, have been observed to also amplify *B. miyamotoi*, a relapsing fever spirochete not part of the BBSL species complex (unpublished own data). As such, samples that were not screened for specific *Borrelia* species have to be considered as carrying *Borrelia* spp. rather than BBSL. Consequently, we refer to *Borrelia* spp. when collectively discussing all detections of *Borrelia* made in the current study. The assay protocols, primers, and probes have been reported previously [9, 12].

### 2.3. Statistical Analysis

Due to the low number of nymphs (*n* = 68), analyses regarding differences in pathogen occurrence between ticks removed from dogs and cats and between the two tick species were made only for adults (*n* = 6006). We used generalized linear mixed models (GLMM) with binary distributions and logit link functions to estimate the probabilities of carrying each pathogen (i.e., receiving the value “1”) and included administrative region (site of collection) as a random variable to control for spatial variation in the data. The administrative region of Åland Islands was dropped from analyses due to only one sample originating from the area, leading to problems with confidence limits and complete separation in models [55]. We ran single analyses for each pathogen, including tick species, host species, and an interaction term (tick × host) as fixed effects in the models.

All the models were run with the GLIMMIX procedure of SAS v. 9.4. using residual pseudo likelihood estimation. Denominator degrees-of-freedom were approximated for the fixed effects by the Kenward–Roger method. Multiple, a posteriori, pairwise comparisons for differences of the estimated marginal means (i.e., ls-means in SAS) were adjusted by the Tukey–Kramer method.

## 3. Results

Out of the 14,889 ticks removed from dogs and cats, 14,747 (99%) were adults, 140 (0.9%) nymphs, and 2 (0.1%) larvae. Out of these, 11,884 (79.8%) ticks were identified as *I. ricinus* and 3005 (20.2%) as *I. persulcatus*. While not documented in a way that allows further analysis, it was noted during the processing of the crowdsourced samples that ticks removed from dogs and cats were often in visible stages of engorgement, indicating considerable times of attachment prior to removal [56].

A total of 4423 ticks collected from dogs (4395 adults and 28 nymphs) and 1658 ticks collected from cats (1619 adults and 39 nymphs) were screened for the presence of the listed TBPs (altogether 6081 samples). Out of these, 3697 were identified as *I. ricinus* (3684 adults and 13 nymphs) and 2355 as *I. persulcatus* (2322 adults and 33 nymphs). Overall, 35.0% ± 1.2% (95% confidence limits) of adults and 20.9% ± 9.7% of nymphs carried at least one of the screened pathogens. Prevalence rates in adult ticks were 26.2% ± 1.1% for *Borrelia* spp., 9.3% ± 0.7% for *Rickettsia* spp., 1.1% ± 0.3% for *A. phagocytophilum*, 1.1% ± 0.3% for TBEV, 0.6% ± 0.2% for *N. mikurensis*, and 0.4% ± 0.1% for *Babesia* spp. (see Table S1 for nymph prevalence). *Bartonella* spp. and *F. tularensis* were not detected in adults or nymphs. Prevalence rates in adults by tick and host species are reported in Table 1. All the detected pathogens were observed from ticks removed from both host species as well as both tick species. Co-infections with two or more pathogens were detected in 224 ticks (3.7 ± 0.4%).

Differences in the probability of carrying *Borrelia* spp. were detected both between ticks collected from dogs (0.25 [0.21–0.29]; 95% confidence interval) and cats (0.21 [0.17–0.25]) (*F*_1, 6002_ = 7.26, *p*=0.007), as well as between *I. ricinus* (0.25 [0.21–0.29]) and *I. persulcatus* (0.20 [0.17–0.25]) (*F*_1, 528_ = 5.34, *p*=0.020). The probability of carrying *A. phagocytophilum* was higher for *I. ricinus* (0.015 [0.009–0.024]) than *I. persulcatus* (0.005 [0.002–0.013]) (*F*_1, 213_ = 4.52, *p*=0.030). Likewise, there appeared to be a trend towards *I. ricinus* having a higher probability of carrying *Rickettsia* spp. (0.11 [0.08–0.14]) than *I. persulcatus* (0.08 [0.06–0.11]) (*F*_1, 537_ = 3.48, *p*=0.060). Finally, the probability of carrying *N. mikurensis* appeared to be higher for ticks removed from dogs (0.007 [0.004–0.010]) than for ticks removed from cats (0.003 [0.001–0.007]) (*F*_1, 6004_ = 3.66, *p*=0.056). No other differences between tick or host species were observed. All interaction terms between tick and host species were non-significant (*p* > 0.05). Please observe that values reported above are predicted probabilities obtained from the statistical models, which may differ from prevalence rates reported in Table 1.

**Figure 2:**
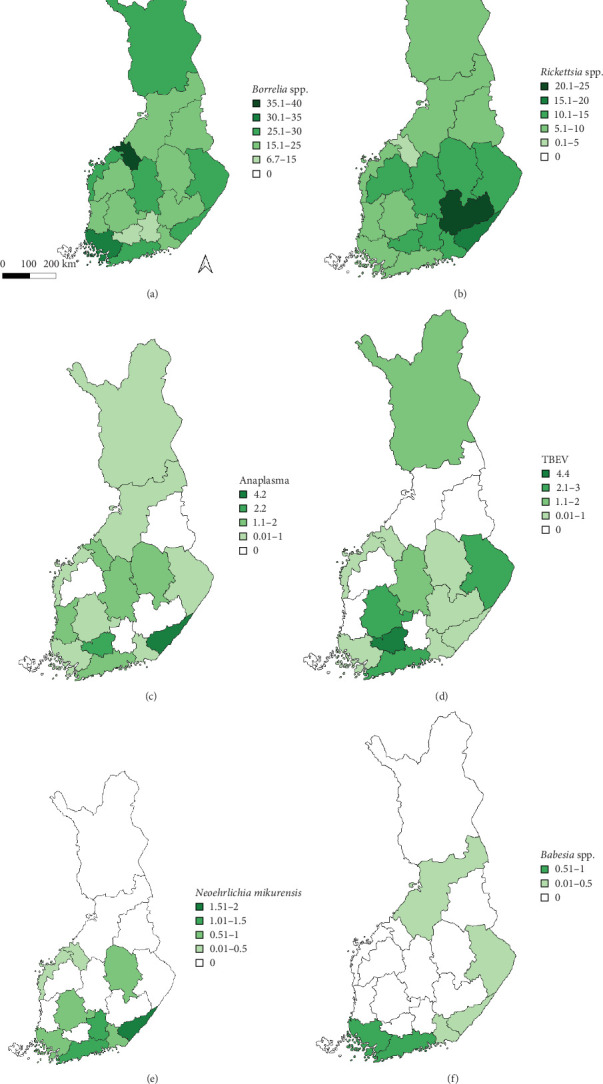
Prevalence of (A) *Borrelia* spp., (B) *Rickettsia* spp., (C) *A. phagocytophilum*, (D) tick-borne encephalitis virus, (E) *N. mikurensis*, and (F) *Babesia* spp. in ticks removed from dogs and cats by administrative region.

**Figure 3:**
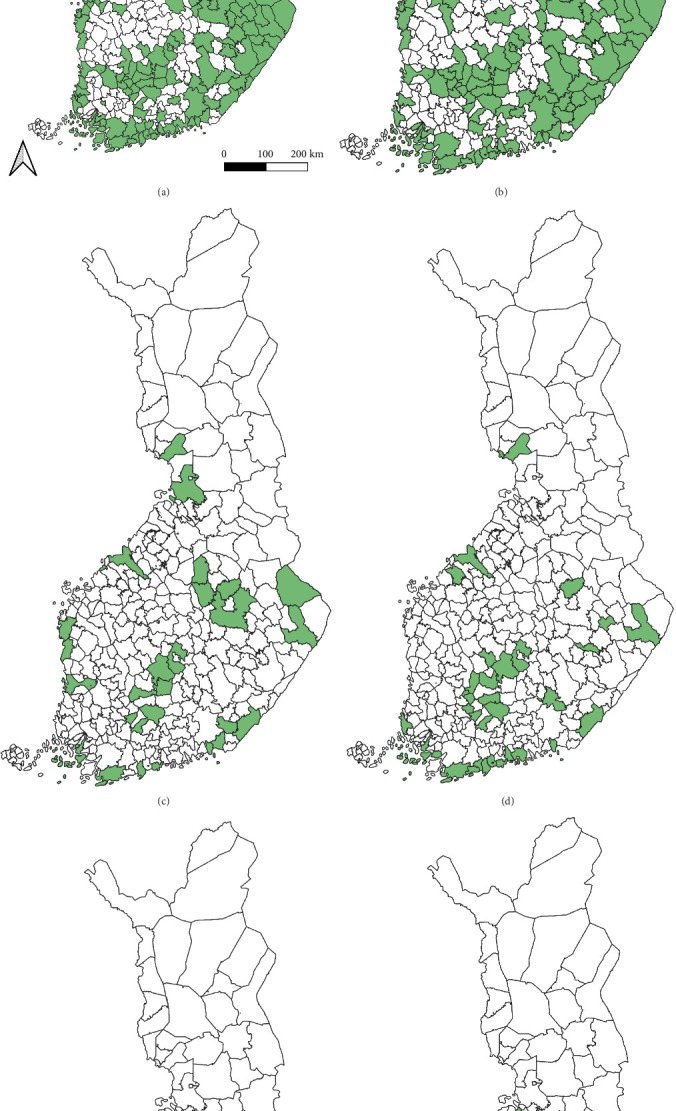
Presence of (A) *Borrelia* spp., (B) *Rickettsia* spp., (C) *A. phagocytophilum*, (D) tick-borne encephalitis virus, (E) *N. mikurensis*, and (F) *Babesia* spp. in ticks removed from dogs and cats by municipality. Municipalities from which the pathogen was detected from are colored green.

Regarding the spatial occurrence and prevalence of TBPs, varying sample numbers and the rarity of certain pathogens translated into wide confidence intervals for many administrative regions and pathogens (Table S2; Figure 2). For this reason, prevalence estimates were not made on the municipality level, only presence/absence classification (Figure 3). Furthermore, we refrained from statistical analysis of the prevalence data at the administrative region scale due to these factors, instead focusing on observing trends from the raw data, which is provided in Table S2. It should be noted that smaller sample sizes in certain administrative regions may influence the observed prevalence, especially for rare pathogens. *Borrelia* spp. and *Rickettsia* spp. were the only pathogens that were detected from tick samples from every Finnish administrative region (Figure 2). While there was variation in estimated *Borrelia* prevalence, no apparent spatial trends in occurrence could be identified (Figure 2A; Table S2). For *Rickettsia*, prevalence appeared to be highest in the central and eastern parts of Finland (Figure 2B). *Borrelia* and *Rickettsia* were detected from 127 and 120 municipalities (out of a total of 309), respectively (Figure 3A, B). The highest estimated prevalence rates for *A. phagocytophilum*, *N. mikurensis*, and *Babesia* were observed in southern administrative regions (Figure 2C,E,F). These three pathogens were detected from 29, 22, and 12 municipalities, respectively (Figure 3C,E,F). TBEV occurrence was mostly focused around the coastline and archipelago of the Baltic Sea and large inland lakes (Figure 2D). TBEV was detected from 33 municipalities (Figure 3D).

## 4. Discussion

Dogs and cats are commonly exposed to ticks and TBPs in Finland. The crowdsourcing study conducted in 2015, from which the utilized tick samples originated, showed that out of the 16,971 tick samples paired with host data, 55.9% were collected from dogs, whereas a further 27.6% were from cats [4]. In the current study, we screened a subset of 6081 of these samples for the TBPs most commonly detected from questing ticks in Finland, revealing that ticks infesting dogs and cats also frequently carry pathogens. During the processing of the crowdsourced samples, it was noted that ticks removed from dogs and cats were often in visible stages of engorgement, indicating considerable times of attachment prior to removal [56]. The same observation was made from ~5700 pictures uploaded during 2021 to the tick surveillance website Punkkilive (www.punkkilive.fi), which showed ticks in visible stages of engorgement in 42.6% of pictures (with 59.5% of all observations reported from dogs or cats) [5]. These findings conform to reports of long attachment times of ticks removed from dogs and cats from Germany and Austria [57] and suggest that protection of dogs and cats from ticks is regularly insufficient. Consequently, even pathogens that require a longer time to transfer from an infected tick to the host (such as *Borrelia* spp. and *A. phagocytophilum*) likely frequently have enough time to transfer when feeding on dogs or cats [58, 59].

One curiosity regarding the data of ticks removed from cats and dogs (and humans) in crowdsourcing campaigns are the mirrored life stage ratios compared to wild populations. In the crowdsourced data set used here, 99% of ticks removed from cats and dogs were adults [4]. Likewise, in a study conducted at several Finnish veterinary clinics, 98.5% of ticks removed from dogs and cats were adults [6]. Finally, in ~5700 pictures uploaded to the Punkkilive-website in 2021, adult ticks could be identified in 55.8%, whereas nymphs only in 14% [5]. These results starkly contrast observations regarding *I. ricinus* from field surveys, where larvae and nymphs have consistently been observed to be more numerous than adults [7, 8, 18, 60]. However, for the somewhat less common *I. persulcatus*, more adults are typically collected than nymphs, indicating some differences in behavior between these species [7, 12, 61]. While there is a well-documented preference for large hosts for adult ticks and medium to small hosts for juvenile tick life stages, the almost complete lack of juveniles observed here is nevertheless peculiar. Dogs come in many different sizes, only some of which may be classified as “large hosts”—several dog breeds are medium, with also high variation in, for example, fur length and undercoat characteristics. Thus, several breeds should be suitable hosts for at least nymphs. Indeed, dogs have previously been identified as common sources of blood meals for larvae based on bloodmeal analysis [62]. Similarly, cats are likely to encounter juvenile ticks, not only because of their size but also their stalking behavior when outdoors. Likewise, the division of tick life stages to hosts of different sizes is in reality not clear-cut, with the biology of the host itself also being highly relevant [63]. For example, juvenile ticks are commonly detected feeding on several species of deer [64–66], animals classified as “large hosts.” Furthermore, on an island in Finland, up to 37% of questing nymphs at a study site were observed to have fed on deer as larvae [53], indicating that larvae also commonly utilize these large hosts. Consequently, we would have expected a significant proportion of juvenile ticks among the samples from dogs and cats. The most likely explanation for this phenomenon is that people (including veterinarians) do not find or detect the small juvenile ticks from the fur of the animals. It is also possible that a large portion of juvenile ticks are destroyed beyond recognition during removal, so citizen scientists have nothing left to send. Dogs and cats have generally been seen as dead-end hosts for ticks, but this observation calls to question whether they may in fact be contributing to tick population upkeep by feeding juvenile ticks. In any case, if nymphs feeding on dogs and cats are routinely missed, the exposure of these animals to TBPs may be even greater than has been observed.

The prevalence rates of the analyzed TBPs generally conform to values reported from questing ticks all over Finland [7, 8, 10–12, 18, 23, 46–49, 51, 60, 67]. *Borrelia* spp. were the most commonly detected pathogens, as also observed in the aforementioned field surveys. Compared to a recent study analyzing ticks removed from dogs and cats at veterinary clinics in Finland [6], we observed higher prevalence of *Borrelia* and TBEV and lower prevalence of *Babesia* and *A. phagocytophilum*. While some of the ticks analyzed here were feeding on the same hosts and/or had engorged already on the host, the overall prevalence rate in adults was in line with field surveys. In fact, higher prevalence rates have been observed in questing ticks in several field study sites [12, 18], indicating that collecting feeding ticks from dogs and cats does not inflate prevalence estimates to any noticeable degree. However, it should be noted that varying stages of engorgement may also reduce TBP detection numbers to an unknown degree—as may be the case here.

Regarding the spatial occurrence of pathogens, uneven geographical distribution of samples hindered analyses. For two administrative regions (out of 18), less than 20 samples were available, whereas for another three regions, only 40–70 samples were available. In addition, the rarity of several pathogens created wide confidence intervals despite adequate sample sizes. While the issue of rare pathogens and confidence intervals is difficult to overcome (e.g., we analyzed 1003 samples from Southwest Finland but still produced wide confidence intervals for rare *A. phagocytophilum*, *Babesia* spp., and TBEV), future collection efforts should specifically target administrative regions that have been identified as providing too few samples in order to mitigate error due to inadequate sample sizes. Targeted media and citizen science campaigns could be organized to specifically activate citizens in these areas.

*Rickettsia* spp. and *A. phagocytophilum* were detected equally from ticks removed from cats and dogs, but the probability of carrying the pathogens appeared to be slightly higher in *I. ricinus* than *I. persulcatus*. However, these interspecific differences are unlikely to have any major biological significance. While several species of *Rickettsia* have been reported to cause rickettsiosis in cats and dogs [20, 68], diagnosed feline or canine cases caused by the species vectored by ticks in Finland (*R. helvetica*, *R. monacensis*, *Candidatus* R. tarasevichiae) [9] have not been reported. The same is true regarding human cases, despite the relatively high prevalence in ticks in Finland [7, 8, 10, 11, 18]. As such, tick-mediated rickettsiosis appears to currently not have much importance from a veterinary or medical point-of-view in Finland. Regarding *A. phagocytophilum*, diagnosed cases have been reported from cats [69]—also in Finland [25]—but rarely. For dogs, seropositivity between 4% and 45% has been reported locally [17, 70], but diagnosed cases nevertheless appear rare [71]. In any case, the high exposure of dogs and cats to ticks, as well as the potential increase in exposure due to increasing tick numbers, means that anaplasmosis should be retained as a possible diagnosis.

*N. mikurensis* was mostly found from ticks collected from the southern parts of Finland and in low prevalence. While the timing of the arrival of this pathogen to Finland and the rate of its spread therein are unknown, its initial establishment was detected in 2015 on an island in southwestern Finland, where tick and TBP surveillance had been ongoing since 2012 [10]. However, several positive samples from across Finland were likewise already found from the crowdsourced samples used in the current study, which were also collected in 2015 [9]. It is likely that sporadic introductions have been and are taking place, with climate change potentially creating novel areas suitable for circulation of the pathogen. The occurrence of *N. mikurensis* seems to be focused on southern Finland [7], where shrews and deer were recently identified as potential additional sources of *N. mikurensis* infections in *I. ricinus* nymphs (in addition to voles) [53]. Particularly the densities of deer (white-tailed deer, *Odocoileus virginianus*, and roe deer, *Capreolus capreolus*) are highest in the southern parts of Finland, potentially enhancing enzootic cycles of the pathogen locally. While no feline or canine cases of neoehrlichiosis have been reported from Finland thus far, the first human case was identified in 2023 [72]. The status of *N. mikurensis* as an emerging zoonotic pathogen means that it should be kept under surveillance.

The prevalence of *Babesia* spp. was low, conforming to previous reports regarding questing ticks, where the pathogen has been reported consistently but in low prevalence [7, 8, 10, 18, 51]. Feline and canine babesiosis have been reported from several countries in Europe, but cases have generally been linked to species of *Babesia* that have not been detected from ticks in Finland [31, 34, 73]. Indeed, while some cases of canine babesiosis have been reported from Finland as well, these were shown to be *Ba. canis* infections acquired from elsewhere in Europe [33]. As far as we are aware, no infections acquired within Finland have been reported [74, 75]. As with *N. mikurensis*, the occurrence of *Babesia* in ticks appears to be centered around areas with high deer densities, which is not surprising, given their likely role as reservoirs of the locally present species [76].

Finally, TBEV was detected from ticks removed from both cats and dogs. The prevalence rates observed in the current study were similar to rates reported from questing ticks in Finland [50, 52, 67], but somewhat higher than those reported recently from ticks removed from dogs and cats at veterinary clinics in Finland [6]. While the prevalence of TBEV is quite low and occurrence highly focal, the high numbers of tick encounters for cats and dogs increase the chances of exposure to the virus, with chances further amplified by increasing tick densities. In addition to increasing tick numbers, novel TBEV foci have been reported from Finland during the past few decades, including from within the capital region, where human and canine activity is high [37, 38]. With more ticks and TBEV foci, the exposure rates of dogs and cats to infected ticks are likely to keep increasing. While TBE is apparently asymptomatic in cats, even fatal cases have been reported for dogs [40]. Consequently, efforts to increase tick knowledge among the public and to protect dogs from ticks need to be continued.

## 5. Conclusions

This study shows that dogs and cats in Finland are frequently exposed to ticks carrying TBPs capable of causing infections. Furthermore, *Borrelia* spp. appear to be present in all the areas with the highest human and pet activity, indicating that most pets making outdoor visits in Finland are under risk of *Borrelia* infection. Future work should encompass the identification of detected pathogens to the species level in order to, for example, identify potential novel threats. As the risk of TBDs may be expected to continue to rise due to the warming climate, further efforts should be undertaken to increase tick knowledge and awareness among pet owners, including by highlighting that there is a tangible risk of tick exposure up to roughly 66° northern latitude. Likewise, methods for protecting the animals from ticks should be promoted and further developed. Finally, the ticks removed from dogs and cats were almost exclusively adult ticks, despite juvenile life stages being more numerous in nature. This raises questions about the ability of dogs and cats to successfully feed and disperse juvenile ticks. The contribution of dogs and cats in the upkeep of tick populations requires further study, particularly in urban areas where these animals may visit specific green spaces several times daily, giving engorged ticks ample opportunities to return to nature.

## Figures and Tables

**Table 1:** Positive sample numbers and prevalence (%) of analyzed pathogens (with 95% CL) in adult ticks by host and tick species.

Pathogen	Positive samples per category (prevalence and 95% CL)			
	Host (no. analyzed)		Tick species (no. analyzed)	
	Dog (4388)	Cat (1618)	*I. ricinus* (3684)	*I. persulcatus* (2322)
*Borrelia* spp.	1194^a^ (27% ± 1%)	379^a^ (23.4% ± 2%)	1002^b^ (27.2% ±1.4%)	571^b^ (24.6% ± 1.8%)
*A. phagocytophilum*	41 (1% ± 0.3%)	26 (1.6% ± 0.6%)	54^b^ (1.5% ±0.4%)	13^b^ (0.6% ± 0.3%)
*Rickettsia* spp.	396 (9% ± 0.8%)	161 (10% ± 1.5%)	388 (10.5% ± 1%)	169 (7.3% ± 1.1%)
*Babesia* spp.	19 (0.4% ± 0.2%)	3 (0.2% ± 0.2%)	20 (0.5% ± 0.2%)	2 (0.1% ± 0.1%)
*N. mikurensis*	34 (0.8% ± 0.3%)	5 (0.3% ± 0.3%)	33 (0.9% ± 0.3%)	6 (0.3% ± 0.2%)
TBEV	46 (1% ± 0.3%)	18 (1.1% ±0.5%)	45 (1.2% ±0.4%)	19 (0.8% ± 0.4%)

*Note*: Only ticks with positive species identification were used in the table. No detections of *Bartonella* spp. or *F. tularensis* were made.

Abbreviation: TBEV, tick-borne encephalitis virus.

^a^Statistically significant (*p*  < 0.05) differences between host species. See text for details.

^b^Statistically significant (*p* < 0.05) differences between tick species. See text for details.

## Data Availability

The data that support the findings of this study are available from the corresponding author upon reasonable request.

## Supporting Information

Supporting Information Table S1. Nymph samples (*n* = 67) positive for screened pathogens with prevalence and 95% confidence limits. Table S2. Numbers of positive samples and prevalence rates (with 95% confidence limits) of different pathogens in ticks removed from dogs and cats on the administrative region level. See Figure 1 for geographical locations of administrative regions.
