# Supplementary material for: Ticks and Tick-Borne Pathogens Encountered by Dogs and Cats: A North European Perspective
Source: Transbound Emerg Dis. 2025 Jun 4;2025:5574554. doi: 10.1155/tbed/5574554 (PMC12158588; doi:10.1155/tbed/5574554)
Supplement: Supporting Information — Table S1. Nymph samples (n = 67) positive for screened pathogens with prevalence and 95% confidence limits. Table S2. Numbers of positive samples and prevalence rates (with 95% confidence limits) of different pathogens in ticks removed from dogs and cats on the administrative region level. See Figure 1 for geographical locations of administrative regions. [file 5574554.f1.docx]

**Ticks and tick-borne pathogens encountered by dogs and cats: a North European perspective**

Jani J. Sormunen, Eero J. Vesterinen, Tero Klemola

**Supplementary materials**

Contents

[Table S1. 1](#_Toc171498221)

[Table S2. 2](#_Toc171498222)

# Table S1.

Nymph samples (*n*=67) positive for screened pathogens with prevalence and 95% confidence limits.

| Pathogen | Nymph samples (*n*=67) positive for pathogen (prevalence ±95% confidence limits) |
| --- | --- |
| *Borrelia* spp. | 8  (11.9 ± 7.8) |
| *Rickettsia* spp. | 6  (9 ± 6.8) |
| *Anaplasma phagocytophilum* | 1  (1.5 ± 2.9) |
| Tick-borne encephalitis virus | 1  (1.5 ± 2.9) |
| *Neoehrlichia mikurensis* | 0 |
| *Babesia* spp. | 0 |
| *Bartonella* spp. | 0 |
| *Francisella tularensis* | 0 |

# Table S2.

Numbers of positive samples and prevalence rates (with 95% confidence limits) of different pathogens in ticks removed from dogs and cats on the administrative region level. See Figure 1 for geographical locations of administrative regions.

| Region | Samples | Positive samples (prevalence and 95% confidence interval) | | | | | |
| --- | --- | --- | --- | --- | --- | --- | --- |
|  |  | Bbsl | Rspp | Ana | Neo | Babe | TBEV |
| Southwest Finland (SF) | 1003 | 315  (31 ±3) | 74  (7.4 ±1.6) | 10  (1 ±0.6) | 9  (0.9 ±0.6) | 9  (0.9 ±0.6) | 7  (0.7 ±0.5) |
| Uusimaa (UM) | 892 | 249  (27.9 ±3) | 78  (9 ±1.9) | 10  (1.1 ±0.7) | 11  (1.2 ±0.7) | 9  (1 ±0.7) | 20  (2.2 ±1) |
| Kymenlaakso (KL) | 256 | 47  (18.4 ±5) | 22  (8.6 ±3.4) | 2  (0.8 ±1.1) | 0 | 0 | 2  (0.8 ±1.1) |
| Kanta-Häme (KH) | 45 | 3  (6.7 ±7) | 6  (13 ±10) | 1  (2.2 ±4.3) | 0 | 0 | 2  (4.4 ±6) |
| Päijät-Häme (PH) | 70 | 6  (8.6 ±6.6) | 9  (13 ±7.8) | 0 | 1  (1.4 ±2.8) | 0 | 0 |
| South Karelia (SK) | 288 | 81  (28.1 ±5.2) | 53  (18.4 ±5) | 12  (4.2 ±2.3) | 5  (1.7 ±1.5) | 1  (0.3 ±0.7) | 1  (0.3 ± 0.7) |
| Satakunta (SA) | 62 | 11  (17.7 ±9.5) | 4  (6.5 ±6.1) | 1  (1.6 ±3.1) | 0 | 0 | 0 |
| Pirkanmaa (PM) | 561 | 98  (17.5 ±3.1) | 43  (7.7 ±2.2) | 1  (0.2 ±0.3) | 4  (0.7 ±0.7) | 0 | 14  (2.5 ±1.3) |
| South Savo (SS) | 108 | 22  (20.4 ±7.6) | 23  (21 ±7.7) | 0 | 0 | 0 | 1  (0.9 ±1.8) |
| Central Finland (CF) | 161 | 47  (29.2 ±7) | 22  (13.7 ±5) | 2  (1.2 ±1.7) | 0 | 0 | 2  (1.2 ±1.7) |
| North Karelia (NK) | 314 | 82  (26.1 ±4.9) | 45  (14.3 ±4) | 1  (0.3 ±0.6) | 0 | 1  (0.3 ±0.6) | 7  (2.2 ±1.6) |
| Ostrobothnia (OB) | 480 | 122  (25.4 ±3.9) | 32  (6.7 ±2.2) | 3  (0.6 ±0.7) | 2  (0.4 ±0.6) | 0 | 1  (0.2 ±0.4) |
| South Ostrobothnia (SO) | 14 | 3  (21 ±21.5) | 2  (14 ±18) | 0 | 0 | 0 | 0 |
| North Savo (NS) | 709 | 153  (21.6 ±3) | 75  (10.6 ±2) | 8  (1.1 ±0.8) | 4  (0.6 ±0.6) | 0 | 2  (0.3 ±0.4) |
| Central Ostrobothnia (CO) | 428 | 157  (36.7 ±4.6) | 14  (3.3 ±1.7) | 6  (1.4 ±1.1) | 1  (0.2 ±0.5) | 0 | 3  (0.7 ±0.8) |
| North Ostrobothnia (NO) | 440 | 95  (21.6 ±3.8) | 36  (8.2 ±2.6) | 1  (0.2 ±0.4) | 0 | 1  (0.2 ±0.4) | 0 |
| Kainuu (KA) | 12 | 2  (16.7 ±21) | 1  (8.3 ±16) | 0 | 0 | 0 | 0 |
| Lapland (LL) | 238 | 70  (29.4 ±5.8) | 12  (5 ±2.8) | 1  (0.4 ±0.8) | 0 | 0 | 4  (1.7 ±1.6) |
